# Human papilloma virus infection and mismatch repair protein expression in sebaceous neoplasms of the genital area

**DOI:** 10.1111/his.70078

**Published:** 2025-12-28

**Authors:** Katharina Wiedemeyer, Zainab Al‐Shamma, Martin Köbel, Ingrid Ferreira, Paul W. Harms, Thomas Brenn

**Affiliations:** ^1^ Department of Pathology and Laboratory Medicine University of Calgary Calgary Alberta Canada; ^2^ Department of Pathology University of Michigan Ann Arbor Michigan USA; ^3^ Experimental Cancer Genetics Wellcome Sanger Institute, Wellcome Genome Campus Cambridge UK; ^4^ Arnie Charbonneau Cancer Institute, Cumming School of Medicine University of Calgary Calgary Alberta Canada

**Keywords:** genital, human papilloma virus, mismatch repair, sebaceoma, sebaceous adenoma, sebaceous carcinoma, sebaceous skin tumours, vulva

## Abstract

**Aims:**

This study aimed to investigate the clinical and histopathological features of sebaceous tumours of the genital area and their association with human papilloma virus (HPV) infection and mismatch repair (MMR) protein deficiency.

**Methods and Results:**

Ethical approval was obtained, haematoxylin and eosin (H&E)‐stained sections were reviewed, and immunohistochemistry (IHC) for p16, p53, mismatch repair proteins and HPV RNA‐in‐situ hybridization or HPV genotyping were performed. Clinical follow‐up was retrieved from patient records.

Six tumours presented in adulthood (median: 69; range: 48–73 years; M:F = 2:1) and were located on the penis, mons pubis and labia majora (median size 1.3 cm). There were four sebaceous carcinomas, one sebaceoma and one sebaceous adenoma. Three of four sebaceous carcinomas showing an overlying in‐situ component were positive for high‐risk HPV‐ISH or subtypes, p16 block positive and p53 wild‐type by IHC. The sebaceous adenoma showed loss of mismatch repair proteins and was associated with Muir–Torre syndrome (MTS), while all remaining tumours showed intact MMR protein staining, were negative for HPV, and p16 and p53 wildtype by IHC. The patient with MTS died of oesophageal adenocarcinoma; all other patients were alive without recurrences (median follow‐up: 16 months, range 7–60 months).

**Conclusions:**

In conclusion, the study emphasizes a pathogenetic role of HPV in genital sebaceous carcinomas that typically present with an in‐situ component.

## Graphical abstract

This study establishes the role of high‐risk HPV infection in cutaneous sebaceous carcinoma of the genital area. An intraepithelial component in a subset of cases poses a challenge to distinguish the entity from squamous cell carcinoma in situ. Rarely, sebaceous neoplasia of the genital area is associated with Muir–Torre syndrome.

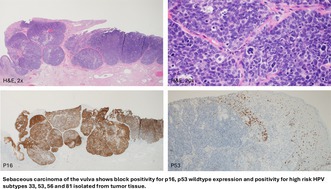

**Abbreviations**
CK7: cytokeratin 7
EMA: epithelial membrane antigen
FFPE: formalin‐fixed, paraffin‐embedded
H&E: haematoxylin and eosin
HPV: human papilloma virus
HSIL: high‐grade squamous intraepithelial lesion
IHC: immunohistochemistry
LSIL: low‐grade squamous intraepithelial lesion
MALDI: matrix‐assisted laser desorption/ionization
MMR: mismatch repair protein
MTS: Muir–Torre syndrome
TOF: time‐of‐flight
UV: ultraviolet

## INTRODUCTION

Cutaneous tumours with sebaceous differentiation most commonly involve the head and neck area and are classified into sebaceous adenoma, sebaceoma and sebaceous carcinoma.^1^ Sebaceous adenoma and sebaceoma are benign tumours and behave indolently. Sebaceous adenoma is composed of lobules of mature sebocytes without atypia or increased mitotic activity, whereas sebaceomas show more than 50% immature, basaloid cells that are mitotically active and may show mild atypia. Sebaceous carcinoma has potential for recurrence, metastasis and mortality, and comprises lobules or nodules of atypical pleomorphic cells with well to poorly recognizable sebaceous differentiation. Sebaceous tumours have been linked to underlying ultraviolet (UV) light exposure signature mutations, defective DNA mismatch repair, paucimutational patterns, mutations in ZNF750, TP53 and RB1 and high‐risk human papilloma virus (HPV) infection.^2^ Patterns of oncogenic drivers differ between (peri)ocular tumours and those arising elsewhere on the skin. Rarely, sebaceous neoplasms are encountered in the genital area despite the high density of sebaceous glands and the exposure to potential HVP infection at this anatomical site.^3^, ^4^, ^5^, ^6^, ^7^, ^8^, ^9^, ^10^, ^11^

The aim of this study is to describe the clinicopathological features of sebaceous neoplasms arising in the genital area and investigate their association with HPV infection and MMR protein deficiency.

## MATERIALS AND METHODS

Ethical approval was obtained from the health research ethics board of Alberta (HREBA.CC‐19‐0379). Haematoxylin and eosin (H&E)‐stained sections of six tumours were retrieved from the departmental files of the Alberta Precision Laboratories, Calgary, Alberta, Canada and of the Department of Pathology, University of Michigan, Ann Arbor, Michigan, USA. The histological features were reviewed, and the following histopathological criteria were documented: tumour circumscription, infiltrative growth, intraepithelial component, cytologic atypia, mitotic activity, necrosis, high‐grade squamous intraepithelial lesion (HSIL) of the adjacent genital epithelium. Immunohistochemistry results were reviewed and additional IHC stains were performed for p53, p16, MLH1, PMS2, MSH2 and MSH6 according to manufacturer's instructions with adequate controls. Wild‐type tumour p53 staining was defined as a mosaic pattering (with mutant patterns being diffuse overexpression or null expression). The source of the antibodies and their dilutions are listed in Table 1. Low‐risk and high‐risk HPV RNA‐in‐situ hybridization and HPV genotyping/subtyping was performed as follows: formalin‐fixed, paraffin‐embedded (FFPE) whole tissue sections of 4‐μm thickness cut freshly from tissue blocks stored at room temperature were deparaffinized; heat‐induced epitope retrieval was performed on a Leica Bond‐III immunostainer using Bond Epitope Retrieval Buffer 2 (ER2) from Leica Biosystems for 15 min at 95°C; the Leica Bond RNAscope Detection kits (Leica Biosystems) were used to detect low‐risk HPV types 6, 11, 40, 42, 43, 44; using the RNAscope 2.5 VS HPV‐LR6 probe (Advanced Cell Diagnostics, cat# 407608) and high‐risk HPV (designed to detect the E6/E7 mRNA from high‐risk HPV types 16, 18, 31, 33, 35, 39, 45, 51, 52, 53, 56, 58, 59, 66, 68, 73, 82; using RNAscope High‐Risk HPV). Sections were incubated with HPV‐LR6 probe/high‐risk HPV probe cocktail for 2 h at 42°C, followed by stepwise amplification of the probe with the AMP1‐AMP6 reagents supplied with the kit. Slides were subsequently counterstained with haematoxylin (5 min) and blued (2 min), all at room temperature on the Leica Bond‐III immunostainer. The MassARRAY (Agena Bioscience) was used for HPV genotyping. The assay is based on PCR and mass array using matrix‐assisted laser desorption/ionization (MALDI) and time‐of‐flight (TOF) mass spectrometry allowing for detection of 19 HPV subtypes (6, 11, 16, 18, 31, 33, 35, 39, 45, 51, 52, 53, 56, 58, 59, 66, 67, 68 and 73). The assay was performed during a 2‐day period: on Day 1, an initial multiple amplification PCR was set up with 2 mL of bio‐banked DNA, followed by a shrimp alkaline phosphatase reaction (which removes the excess nucleotides), followed by an iPLEX pro single base extension PCR (Agena Bioscience); on Day 2, the extension products were desalted with clean resin and loaded into the MassARRAY Dx Nanodispenser RS1000 (Agena Bioscience), transferring the analyte to a spectro chip, which was analysed on the MassARRAY Dx analyser in concordance with the manufacturer's specifications. The mass array assay was performed with an internal glyceraldehyde‐3‐phosphate dehydrogenase control for sample sufficiency and assay performance.

**Table 1:** Lists all antibodies used with their source, incubation times and dilutions

Antibody	Clone	Species	Vendor	Incubation	Dilution
p16	E6H4	Mouse	Ventana	12 min, 36°C	Pre‐dilute
p53	DO‐7	Mouse	Ventana	32 min, 36°C	Pre‐dilute
MLH1	M1	Mouse	Ventana	24 min, 36°C	Pre‐dilute
PMS2	A16‐4	Mouse	Ventana	12 min, 36°C	Pre‐dilute
MSH2	G219‐1129	Mouse	Ventana	12 min, 36°C	Pre‐dilute
MSH6	SP93	Rabbit	Ventana	8 min, 36°C	Pre‐dilute

Clinical data including medical history, genetic test results for microsatellite instability and follow‐up were obtained from patient records.

## RESULTS

### Clinical features

Six tumours were identified affecting four male and two female patients and included four sebaceous carcinomas, one sebaceoma and one sebaceous adenoma. The patient age ranged from 48 to 73 years with a median of 69 years. Three tumours occurred on the penis, two on the labia majora of the vulva and one on the mons pubis (Table 2). The size ranged from 0.6 to 2.7 cm (median 1.3 cm). All tumours were completely excised by primary excision and re‐excision. One male patient with a sebaceous adenoma of the penis had Muir–Torre syndrome (MTS); all remaining patients were *not* diagnosed with a tumour predisposition syndrome. The patient with MTS presented with multiple sebaceous neoplasms (more than 50 in total) consisting mainly of sebaceous adenomas but also including multiple cystic sebaceomas involving various anatomical sites such as the head and neck area, upper and lower extremities, back and the genital area. The patient had a total of six sebaceous adenomas involving the genital area (mons pubis, scrotum and penis). He also developed two keratoacanthomas of the cheek and upper lip. His visceral tumours included a duodenal adenocarcinoma (preceding the skin tumours) and an oesophageal adenocarcinoma presenting simultaneously with the skin neoplasms. Two patients were chronically immunosuppressed (Cases 4 and 6). One female patient (Case 4) with a history of renal transplantation for membranous glomerulonephritis, on tacrolimus and prednisone, and complicated by post‐transplant lymphoproliferative disorder of the B‐cell subtype managed by rituximab, had a history of high‐risk HPV infection of the cervix and vulva. She was previously treated for condylomata acuminata of the cervix and vulva, subsequently developed low‐grade squamous intraepithelial lesion (LSIL) of the cervix, followed by HSIL associated with HPV18 infection of the bilateral labia majora. She underwent recurrent ablative laser surgeries for HSIL until she developed a right‐sided, friable, flesh‐coloured nodule (1 cm) on the labium minor that was diagnosed as an invasive sebaceous carcinoma on histopathology. The other immunosuppressed patient (Case 6) had a diagnosis of cardiac and pulmonary sarcoidosis and was treated intermittently with systemic steroids, methotrexate or azathioprine. He developed a sebaceous carcinoma of the penis, but he also had a history of multiple basal cell carcinomas on the face, trunk and extremities. The remaining three patient histories were uneventful regarding additional tumours and immunosuppression.

**Table 2:** Summary of the clinical, immunohistochemical and HPV status results on sebaceous tumours of the genital area (*n* = 6)

Case	Sex	Age	Site	Size (cm)	Diagnosis	HPV status	P16	P53 pattern	MMRP
1	M	72	Mons pubis	1.5	Sebaceous carcinoma	HPV18	Block +	wildtype	Retained
2	M	73	Penis	2.7	Sebaceous carcinoma	HPV18	Block +	wildtype	Retained
3	F	68	Labium major	2.4	Sebaceous carcinoma	Negative	non‐block +	Wildtype	Retained
4	F	48	Labium minor	1.0	Sebaceous carcinoma	HPV33, 53, 56, 81	Block +	Wildtype	Retained
5	M	67	Penis	0.6	Sebaceous adenoma	Negative	non‐block +	Wildtype	Loss of MSH6
6	M	70	Penis	0.9	Sebaceoma	Negative	non‐block +	Wildtype	Retained

The patient with MTS died of complications of his oesophageal adenocarcinoma; all other patients were alive without recurrences (median follow‐up: 16 months, range 7–60 months).

### Histopathologic features

**Figure 1:**
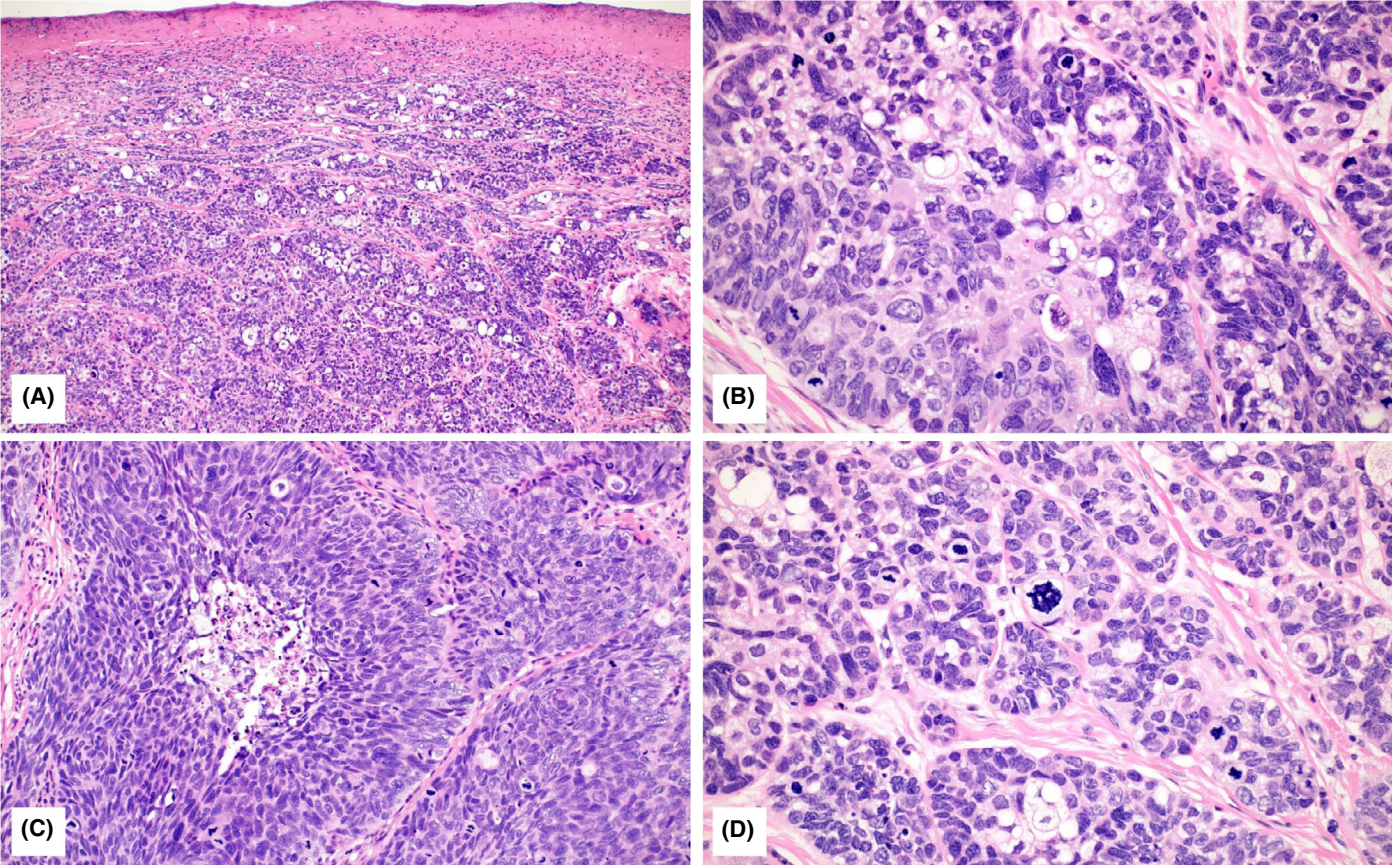
Illustration of sebaceous carcinoma (H&E) of Case 1: the tumour is ulcerated (**A**; 10×) and composed of lobules and sheets of basaloid cells with pleomorphic, oval vesicular nuclei, prominent nucleoli and various amounts of intra‐cytoplasmic lipid vacuoles with foamy appearance (**B**; 40×). Focal necrosis was seen in all cases of sebaceous carcinoma (**C**; 20×). Atypical mitoses are present (**D**; 40×).

**Figure 2:**
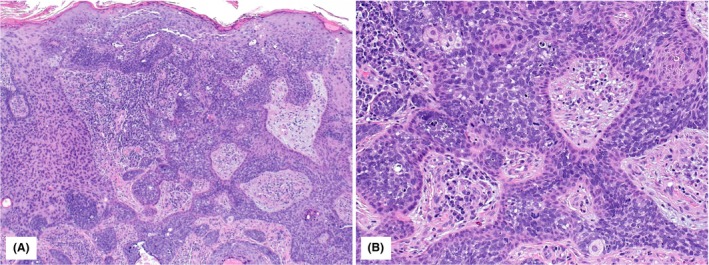
H&E sections of Case 2: the epidermis shows irregular acanthosis and papillomatosis and an extensive sebaceous carcinoma in‐situ component characterized by basaloid tumour cell nests and single basaloid tumour cells with intra‐cytoplasmic lipid vacuoles throughout the full thickness of the epidermis (**A**, 10×; **B**, 40×). Background keratinocyte atypia closely resembles squamous cell carcinoma in situ or high‐grade squamous cell intraepithelial lesion of genital skin.

**Figure 3:**
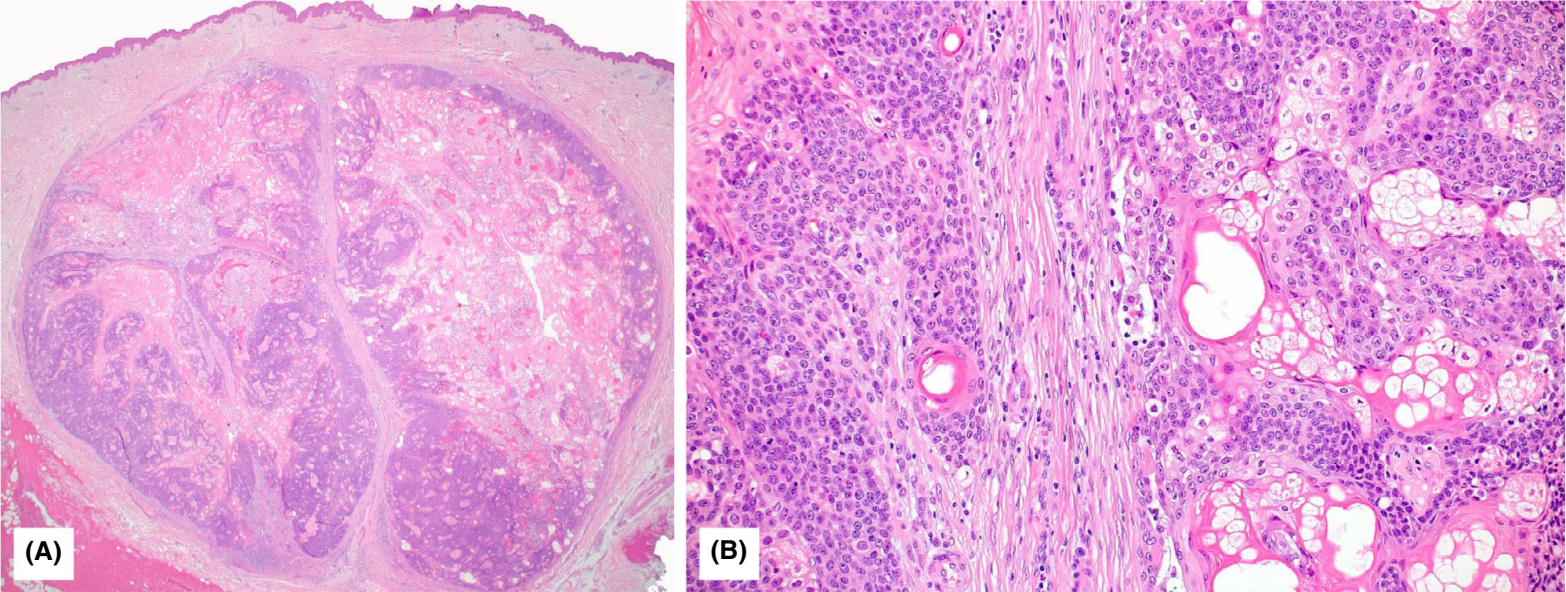
Illustration of Case 6 (H&E): the tumour is well circumscribed, not ulcerated (**A**; 2×) and composed of lobules of basaloid immature sebocytes at the periphery and well‐differentiated sebocytes towards the cystic centre. The mitotic activity is low. No necrosis nor infiltrative growth is seen (**B**; 20×).

The sebaceous carcinomas (Cases 1–4) were poorly circumscribed and composed of basaloid cells with pleomorphic, oval vesicular nuclei, prominent nucleoli and various amounts of intra‐cytoplasmic lipid vacuoles with foamy appearance. The mitotic activity ranged between 3 and 10 mitoses per mm^2^. Focal necrosis was seen in all cases of sebaceous carcinoma (Figure 1A–D). Three of these cases showed an extensive overlying sebaceous carcinoma in‐situ component (Cases 1, 2 and 4) characterized by pagetoid spread of small tumour cell nests and single basaloid tumour cells with intra‐cytoplasmic lipid vacuoles throughout the full thickness of the epidermis (Figure 2A,B). The epidermis revealed irregular acanthosis and papillomatosis with atypia of the keratinocytes closely resembling squamous cell carcinoma in situ or HSIL of genital skin (Figure 2A,B). In comparison, the sebaceous adenoma and the sebaceoma (Cases 5 and 6) were well circumscribed without ulceration. The sebaceous adenoma (Case 5) was composed of several lobules of well differentiated sebocytes with an expanded germinative layer of immature sebocytes at the periphery. The mitotic activity was low. The sebaceoma (Case 6) also showed lobular growth but contained more than 50% basaloid immature sebocytes with low mitotic activity (Figure 3A,B). No necrosis nor infiltrative growth was present neither in Cases 5 nor 6.

### Immunohistochemistry, low‐risk and high‐risk HPV‐ISH results

**Figure 4:**
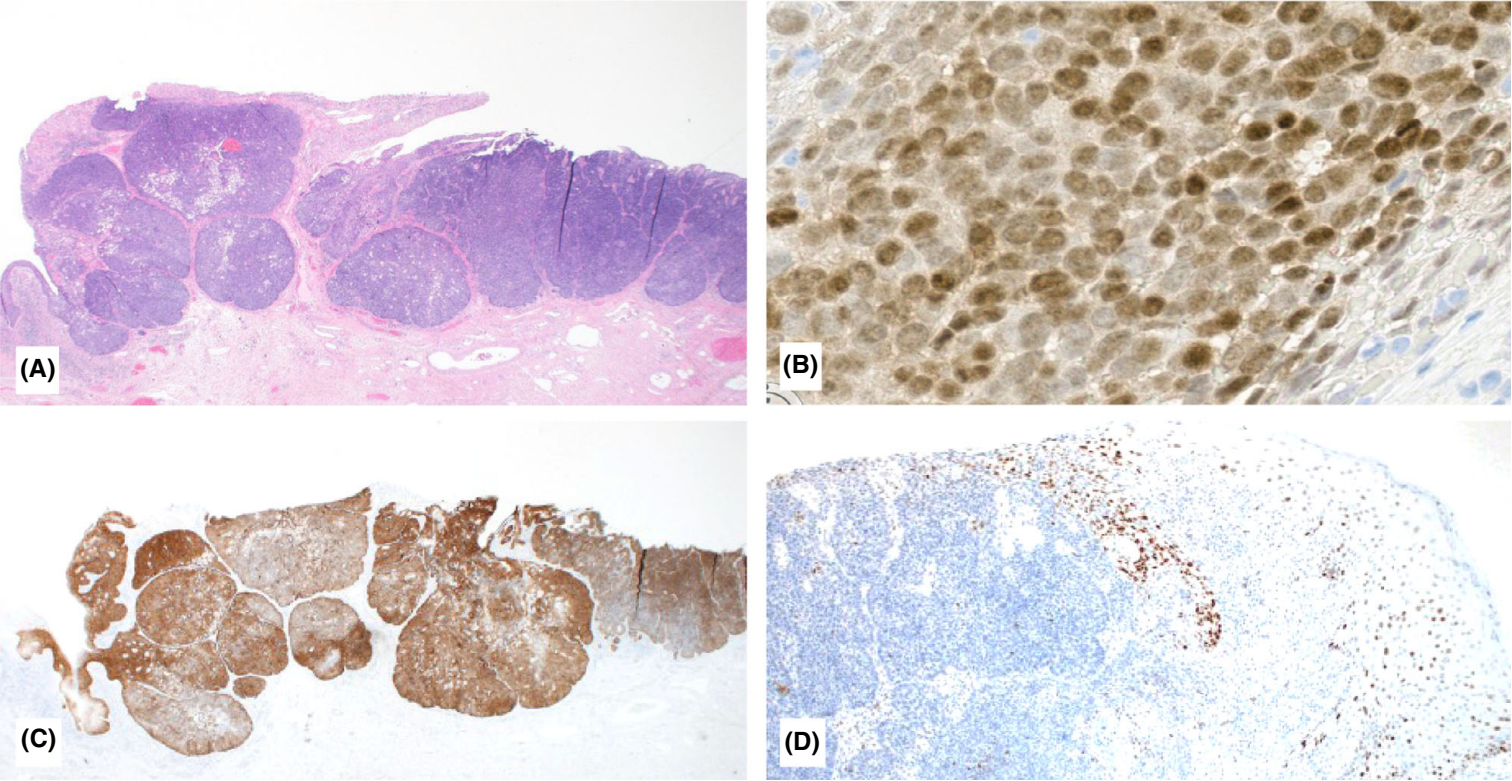
Illustration of Case 4 with IHC findings: H&E sections show an ulcerated, basaloid tumour composed of lobules of atypical cells with various amounts of foamy cytoplasm (1.5×; **A**). The majority of neoplastic cells stain positive for androgen receptor (20×; **B**). P16 is block positive including the in‐situ component of the tumour (1.5×; **C**). P53 shows a wild‐type pattern of expression within the main tumour bulk. The adjacent epidermis shows basal overexpression of p53, consistent with high‐grade squamous intraepithelial lesion (HSIL) (2×; **D**).

The tumours were positive for pancytokeratin, cytokeratin 7 (CK7) and epithelial membrane antigen (EMA) (both available for four of six tumours) and androgen receptor (available for four of six tumours, Figure 4). BerEp4 and Bcl2 showed partial positivity within the more basaloid/immature portion of tumour cells of three sebaceous carcinomas (both available for three tumours). All tumours showed p53 wildtype expression by IHC (Figure 4). All tumours were negative for low‐risk HPV‐ISH. Three of four sebaceous carcinomas were positive for high‐risk HPV‐ISH and/or high rish HPV subtypes. These three tumours demonstrated p16 block positivity (Figure 4), all remaining cases were negative for p16 block expression. The sebaceous adenoma (Case 5) showed loss of MMR protein expression by IHC for MSH6 while all remaining tumours revealed retained MMR protein expression by IHC for MLH1, PMS2, MSH2, MSH6. Of the three sebaceous carcinomas with p16 block expression two showed positivity for HPV subtype 18 and one case (Case 4) revealed positivity for HPV subtypes 33, 53, 56 and 81. The immunohistochemical, high‐risk HPV‐ISH and molecular results are summarized in Table 2.

## DISCUSSION

The exact prevalence of sebaceous neoplasms occurring in the genital area is not known. Less than 15 sebaceous carcinomas of the vulva have been described in the literature to date^12^ and less than 10 sebaceous carcinomas of the penis and scrotum.^11^ Even less data is available on sebaceous adenoma and sebaceoma affecting genital sites.^13^ We conclude that epithelial tumours with sebaceous differentiation are likely underreported and underrecognized. Our study adds to the understanding of the pathogenesis of sebaceous tumours of the genital region. Benign sebaceous tumours may be associated with MTS (a subtype of Lynch syndrome) and sebaceous carcinomas with HPV infection and immunosuppression. Immunosuppression is a well‐known risk factor for the development of sebaceous carcinoma.^2^ TP53 mutations are commonly detected in ocular sebaceous carcinomas^14^ but also occur in sebaceous carcinomas at other cutaneous sites. TP53 mutations are often associated with ZNF750 and RB1 mutations.^2^ The TP53 mutational status in genital sebaceous neoplasms is not known, and routine immunohistochemistry for p53 is usually not performed on sebaceous neoplasms. We therefore do not know the different p53 expression patterns in sebaceous tumours to date. This is very different from vulvar and penile squamous neoplasia that has been extensively studied regarding p53 immunohistochemical expression patterns^15^, ^16^ which can be utilized as a surrogate marker for TP53 mutational status. Six major p53 IHC patterns, two wild‐type patterns (scattered and mid‐epithelial expression), and four mutant patterns (basal overexpression, parabasal/diffuse overexpression, absent and cytoplasmic expression) have been described.^15^ Applying the p53 IHC pattern‐based approach to our case series, p53 IHC showed a wild‐type expression pattern in all tumours.

Our study establishes an etiological link between sebaceous carcinomas in the genital region and high‐risk HPV infection, some occurring in an immune suppressed background. Only recently, HPV infection has been identified as a driver in a subset of ocular sebaceous carcinomas with TP53/RB1 wild‐type status^2^, ^17^ but this link has not been confirmed for cutaneous sebaceous carcinomas arising on other anatomical sites. Vulvar sebaceous carcinoma arising within vulvar HSIL has been described in rare case reports,^3^, ^18^ but HPV infection was not described in these cases. A single case report described a ‘penile squamous cell carcinoma in situ with sebaceous differentiation associated with human papillomavirus type 16’.^19^ The authors did not rule out the possibility that this tumour might represent an undifferentiated intra‐epidermal sebaceous carcinoma composed of primitive germinative sebaceous cells. More recently, two cases of multifocal squamous cell carcinoma in situ with sebaceous differentiation were recognized.^20^ High‐risk HPV‐ISH was also positive in both cases while MMR proteins were retained. Both tumours showed pagetoid spread, and one tumour showed superficial invasion of the dermis. By immunohistochemistry, the neoplastic cells were partially positive for EMA, CK7, p63 and block positive staining with p16. The tumours both recurred over time and required re‐excisions. These cases appear morphologically very similar to the sebaceous carcinomas with extensive intraepithelial components and high‐risk HPV infection in our case series. It is debatable whether in‐situ components represent HPV‐driven primary sebaceous carcinoma versus squamous cell carcinoma with partial sebaceous (trans‐) differentiation. A coexistence of two different cell lineages with HPV driven oncogenesis remains a consideration. In our series, regardless of the classification of the in‐situ component (in cases present), all invasive components show unequivocal sebaceous carcinoma. The p53 wildtype staining pattern can be encountered in both squamous intraepithelial neoplasia and sebaceous carcinoma. It is not known whether the biological behaviour of HPV‐associated sebaceous carcinoma is different from HPV‐driven squamous cell carcinoma in this setting. No treatment guidelines exist for sebaceous carcinoma associated with high‐risk HPV infection.^13^, ^21^ One sebaceous carcinoma in our series was not associated with HPV infection (Case 3). This tumour did not show an in‐situ component nor adjacent HSIL. An in‐situ component might indicate high‐risk HPV infection in sebaceous carcinoma of the genital area according to our limited data.

Sebaceous differentiation is likely underrecognized in genital tumours. The low case number is a limiting factor in this study and shows the need for future projects regarding this topic. The use of immunohistochemical stains such as EMA, androgen receptor and adipophilin in basaloid epithelial tumours can greatly facilitate the detection of sebaceous differentiation.^22^

Sebaceous adenoma and sebaceoma are frequently associated with defective DNA mismatch repair,^23^, ^24^ and it is not surprising that one of the tumours of this case series was associated with MTS. In fact, sebaceous adenomas and sebaceomas arising in unusual anatomical sites should raise suspicion for an underlying tumour predisposition syndrome. Sebaceous neoplasms arising in the genital area have not been described to be especially indicative for MTS, but it is noteworthy that our patient with MTS developed a total of six sebaceous adenomas on the penis and scrotum.

## CONCLUSION

This study establishes the role of high‐risk HPV infection and immunosuppression in cutaneous sebaceous carcinoma of the genital area. An extensive intraepithelial component in a subset of cases poses a challenge to distinguish the entity from squamous cell carcinoma in situ and high‐risk squamous intraepithelial lesions that may be present in the background or precede sebaceous carcinoma. Rarely, sebaceous neoplasia of the genital area is associated with MTS, a subtype of Lynch syndrome.

## Author contributions

**Katharina Wiedemeyer:** study conception and design, data collection, analysis and interpretation of results, manuscript preparation. **Zainab Al‐Shamma:** data collection, analysis and interpretation of results. **Martin Köbel:** data collection, analysis and interpretation of result. **Ingrid Ferreira:** data collection. **Paul W. Harms:** data collection, manuscript preparation. **Thomas Brenn:** data collection, analysis and interpretation of results, manuscript preparation.

## Conflict of interest

The authors have nothing to disclose. The authors have no conflict of interest.

## Consent

Informed consent was not required for this retrospective study because only routinely archived paraffin‐embedded tissue was used without consequences for the patients.

## Data Availability

The data that support the findings of this study are available on request from the corresponding author. The data are not publicly available due to privacy or ethical restrictions.
